# Multisensory and Sensorimotor Integration in the Embodied Self: Relationship between Self-Body Recognition and the Mirror Neuron System

**DOI:** 10.3390/s22135059

**Published:** 2022-07-05

**Authors:** Sotaro Shimada

**Affiliations:** School of Science and Technology, Meiji University, Kawasaki 214-8571, Japan; sshimada@meiji.ac.jp

**Keywords:** sense of ownership, sense of agency, rubber hand illusion, robot hand illusion, full body illusion, mirror neuron system, back projection, functional neuroimaging

## Abstract

The embodied self is rooted in the self-body in the “here and now”. The senses of self-ownership and self-agency have been proposed as the basis of the sense of embodied self, and many experimental studies have been conducted on this subject. This review summarizes the experimental research on the embodied self that has been conducted over the past 20 years, mainly from the perspective of multisensory integration and sensorimotor integration regarding the self-body. Furthermore, the phenomenon of back projection, in which changes in an external object (e.g., a rubber hand) with which one has a sense of ownership have an inverse influence on the sensation and movement of one’s own body, is discussed. This postulates that the self-body illusion is not merely an illusion caused by multisensory and/or sensorimotor integration, but is the incorporation of an external object into the self-body representation in the brain. As an extension of this fact, we will also review research on the mirror neuron system, which is considered to be the neural basis of recognition of others, and discuss how the neural basis of self-body recognition and the mirror neuron system can be regarded as essentially the same.

## 1. Introduction

The embodied self is the self that is situated in the environment “here and now”, and is inseparably intertwined with bodily information. Gallagher [1] proposed the minimal self, which is closely related to the embodied self, as the most basic sense of self that remains when the non-essential aspects of the self are stripped away, and includes both a sense of self-ownership and a sense of self-agency. The sense of self-ownership is the feeling that “this body belongs to me”, and the sense of self-agency is the feeling that “I am the one who caused this action”. The senses of ownership and agency often occur simultaneously, but they can also be separate. For example, unintentional body movements, such as the movement of an arm when someone bumps into you, generate a sense of ownership of your arm, but not a sense of agency of your arm movement. It is possible to say that the sense of ownership is a sense of self arising from bodily multisensory integration, whereas the sense of agency is a sense of self arising from sensorimotor integration regarding intentional movement. Both these senses originate in the body, but are often subconscious and only become conscious when something unusual happens to us (for example, when we stumble over a stone while walking).

We perform various actions in our daily lives, but not all of them are executed consciously. In fact, most of them are performed unconsciously. For example, when we reach a cup of coffee on the table, we do not consider which muscles of our hand to use and how much force to apply. We just think about drinking coffee (or maybe we do not even think about it). Only a handful of pieces of information are processed consciously, and much of the processing at the somatic level in the brain occurs unconsciously.

In contrast, the sense of self is considered to arise when the state of the body is made conscious. For this to occur, information about the body must first be accessible to consciousness. Gallagher defines a “body image” as an internal representation of one’s own body, consisting of perceptions, concepts, and feelings that can be accessed by consciousness [2]. The content of body image does not necessarily correspond to the actual body and may also contain contradictory beliefs. On the other hand, it is thought that a sensory-motor map, which is called a “body schema (motor schema)”, exists in the brain when we perform an action, which indicates what kind of movement is performed in response to a certain sensory input and what kind of sensory feedback is returned by performing a certain movement. The body schema is used to perform appropriate actions in response to the environment without being consciously aware of it. For example, in the previous example of reaching for a coffee cup, the body schema of “reach and grasp” motion is used, but it is impossible to fully report consciously (linguistically) which muscles are used and how.

One might then think that the sense of ownership is related to body image and that the sense of agency is related to body schema. Indeed, the former may be appropriate, but given that the body schema cannot necessarily be made conscious, it is not simple to say that the sense of agency arises from the body schema. This will be addressed later, but the relationship between movement intention and feedback may be rather important for the sense of agency.

## 2. Embodied Self

### 2.1. Sense of Body Ownership and Rubber Hand Illusion

A sense of ownership is usually a feeling one has for one’s own body, but it is also possible to have a sense of ownership for a body-like object other than one’s own body. A typical example is the rubber-hand illusion. The rubber hand illusion is a phenomenon in which a person is given tactile stimuli simultaneously to a fake hand (rubber hand) and the participant’s own hand so that the rubber hand feels as if it were his or her own hand [3]. To experiment with the rubber hand illusion, a rubber hand is first placed on a table and then the participant’s own hand is placed next to it. At this point, a screen is placed between the rubber hand and the participant’s hand, so that the participant cannot directly see his or her own hand. The experimenter then strokes the rubber hand and the participant’s hand simultaneously with a brush. After repeating this for 2–3 min, the participant will feel as if the rubber hand is his or her own hand.

The rubber hand illusion is thought to be a phenomenon in which the rubber hand is incorporated into the body image of the self when visual stimuli given to the rubber hand and tactile stimuli given to the hand are simultaneously input to the brain. The brain interprets the visual body that appears with the sense of touch, a sensation that can only be felt by oneself, as belonging to the “self”, and as a result, the rubber hand is incorporated into the body image and is perceived as one’s own hand.

For the rubber hand illusion to occur, it is important that the visual and tactile stimuli are given in a spatiotemporally consistent manner. In the rubber hand illusion, the two hands (the rubber hand and one’s own hand) are in different positions, so the illusion occurs even if spatial consistency is not strictly established. However, it has been reported that the illusion is significantly weakened when the rubber hand is placed far from the own hand [4] or when it is facing the opposite direction of the own hand [5]; therefore, some degree of spatial consistency is necessary. It has also been repeatedly reported that staggering the timing of the stroking of the two hands prevents the rubber-hand illusion [3]. Experiments examining the temporal consistency between vision and touch necessary for the rubber-hand illusion to occur have shown that the rubber-hand illusion occurs if the delay is within 200–300 ms; however, if the delay is larger than that, the illusion is less likely to occur [6,7]. Thus, these studies indicate that spatiotemporal consistency between senses to a certain degree is necessary for the brain to maintain a self-body image.

The rubber-hand illusion is often examined by means of a subjective evaluation (questionnaire) of the sense of ownership, but it can also be confirmed by a phenomenon called proprioceptive drift. Before and after the experiment, the subject is asked to point to the position of his or her hand (e.g., left hand) from under the desk with the other hand (right hand), while closing his or her eyes. The perceived position of the real hand tends to shift toward the location of the rubber hand, which is called proprioceptive drift. Although it was initially believed that subjective evaluation and proprioceptive drift are correlated, subsequent studies have shown that subjective evaluation and proprioceptive drift do not necessarily correlate [8]. In an experiment conducted by the authors, a group of subjects who were given illusionary stimuli for 3 min confirmed that the rubber-hand illusion occurred in both subjective evaluation (questionnaire) and proprioceptive drift, while a group of subjects who were given only 1 min of stimuli confirmed only proprioceptive drift and no significant results were obtained in subjective evaluation [7]. In fact, there were cases in which proprioceptive drift was observed even when the subjects strongly claimed that the illusion did not occur. In addition, while subjective evaluation changed rapidly and nonlinearly after a visual feedback delay of approximately 200 ms, proprioceptive drift changed linearly with the delay length. These results indicate that subjective evaluation and proprioceptive drift reflect different aspects of the rubber-hand illusion, and it is possible that subjective evaluation is based on body image, whereas proprioceptive drift reflects changes in the body schema.

In addition to these methods, physiological indices can also be used as indicators of the rubber hand illusion. For example, there is a method to examine the subject’s electrodermal response to physically strong stimuli (e.g., hitting with a hammer or sticking a knife into the subject’s hand) applied only to the rubber hand after tactile stimuli have been applied for a sufficiently long time to cause the illusion. The electrodermal response reflects autonomic responses triggered by emotions such as fear or surprise, and it has been shown that the response is significantly greater when illusions are being experienced [4].

The neural mechanisms of the rubber-hand illusion have also been investigated. Ehrsson et al. [5] measured brain activity during the rubber-hand illusion. They compared four conditions combining the orientation of the rubber hand (same or opposite to one’s own hand) and the simultaneity of the stimuli (synchronous or asynchronous). The questionnaire results confirmed that the subjective rubber hand illusion occurred only in the condition in which the rubber hand was facing the same direction as one’s own hand and the stimuli were given synchronously, but not in the other conditions. Brain activity in the ventral premotor cortex correlated with the subjective strength of the rubber hand illusion. Although no correlation was found, differences in activity between the conditions were also observed in the parietal lobe (intraparietal sulcus), with greater activity in the condition in which the rubber-hand illusion occurred. These activities have also been confirmed in more detail in recent studies [9,10]. Brozzoli et al. [11] also reported that activity in the left ventral premotor cortex correlated with subjective evaluation of the rubber hand, and activity in the right inferior parietal lobe (supramarginal gyrus) correlated with the amount of proprioceptive drift. Thus, the sensorimotor network of the ventral premotor and parietal lobes is deeply involved in the rubber-hand illusion.

### 2.2. Sense of Agency and Forward Model

The sense of agency is the feeling that I am the one who controls the movement. The sense of agency is thought to be related to a mechanism of motor control called the forward model (also called the comparator model).

**Figure 1:**
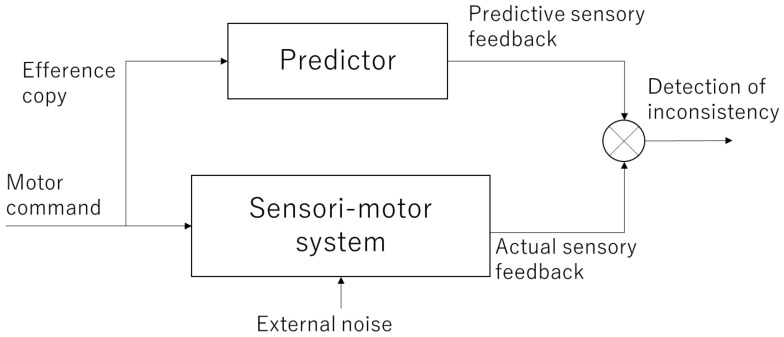
Forward model. The motor commands are sent to the sensori-motor system and the results are returned as actual sensory feedback. Meanwhile, copy information (efference copy) of the motor command is sent to the internal predictor, which generates predictive sensory feedback. By comparing these signals, inconsistency between the intended and actual outcomes is detected.

When performing an action, motor commands are sent from the motor cortex of the brain to muscles. This activates the muscles, which in turn return feedback information, such as changes in body posture and tactile information to the brain. At this time, a copy of the motor command information, called the efference copy, is projected to another area of the brain (most likely the parietal lobe and cerebellum) to check whether the movement was executed as intended by the subject. This efference copy is used to internally predict the sensory feedback that will be returned and compare it with the actual feedback. This mechanism is referred to as the forward model (Figure 1) [12,13,14]. If the prediction of the forward model is correct, it is consistent with the actual sensory feedback, and a sense of agency emerges as it is the movement that one has caused. Conversely, if they are inconsistent, the sense of agency is attenuated or lost.

To experimentally examine the sense of agency, a device in which a camera showing the subject’s hand and a camera showing the experimenter’s hand could be switched was used [15]. The subjects observed the hand shown on the monitor to determine whether it was their own hand or someone else’s hand. If the hands are moving differently, it is immediately obvious that it is someone else’s hand, but if they are moving in the same way, even healthy subjects can make judgment errors. In particular, patients with schizophrenia and patients with parietal lobe damage showed significantly higher error rates, indicating that another person’s hand is their own [15,16]. It is considered that the forward model is malfunctioning in these patients.

According to the forward model, the efference copy is expected to play an important role in generating a sense of agency. Tsakiris et al. [17] conducted an experiment to investigate the effects of efference copy on self-body perception. In the active movement condition, the participants were asked to move their left hand with their right hand using a leverage while their own left hand or someone else’s hand was displayed on the screen. The participant answered significantly more accurately in the active condition than in the passive condition, in which the experimenter manipulated the leverage to distinguish between the self and others. This result indicates that self-motion can be more accurately discriminated when an efference copy is available.

**Figure 2:**
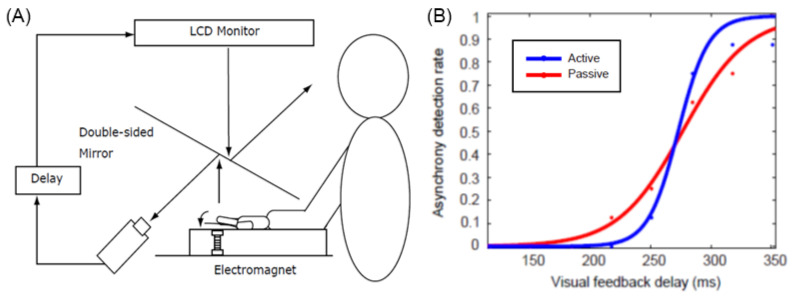
Active and passive vision-movement asynchrony judgement task [14]. (**A**) experimental settings. (**B**) A representative result. The time window for detecting visual feedback delay is nearly equivalent for active and passive movements, while the steepness of the slope is steeper for active movement than for passive movement.

However, the experiment by Tsakiris et al. [17] did not sufficiently control for subtle differences in the movement and timing of the hands of self and others, and it is not clear whether the difference in results between the active and passive conditions is due to the effect of the efference copy alone. Therefore, Shimada et al. [18] developed a device that can move the hand actively or passively using electromagnets and conducted an experiment in which a visual feedback delay of several hundred milliseconds was inserted and presented to the subjects (Figure 2). The results showed that the threshold for delay detection was approximately 230 ms, with no difference between active and passive motion; however, an examination of the slope of the delay detection curve showed that it was significantly steeper in the active condition than in the passive condition. This indicates that there is a time window of approximately 200 ms for delay detection and that the judgment of whether or not a motion is delayed changes abruptly (with strong contrast) in the active condition, whereas it changes more gradually in the passive condition. In other words, the active condition accurately determines whether the delay is within the threshold, whereas the passive condition tends to produce a blurring of judgment. This suggests that the efference copy does not change the width of the time window in the sensorimotor integration of the self, but rather serves to elaborate whether the delay between predicted and actual sensory feedback is within an acceptable range, which leads to discrimination between the self and others.

### 2.3. Implicit Measures of the Sense of Agency

In addition to the subjective questionnaire, there are two other objective measures of the sense of agency: sensory attenuation and intentional binding.

Sensory attenuation is closely related to the forward model described above. When the outcome of an action is as intended, the predictions made by the forward model match the observed outcome, thereby suppressing the response to sensory feedback. It has long been known that the electroencephalographic (EEG) component elicited when listening to a sound stimulus (called the N1 component) is smaller for self-triggered sounds than for externally triggered sounds, which is called N1 suppression [19]. Behavioral experiments have also reported that self-triggered sounds are subjectively weaker than externally triggered sounds [20]. An interesting example is the phenomenon of not feeling tickled when one tickles oneself. This is thought to be due to the forward model, which predicts tactile feedback from one’s own movements, resulting in sensory attenuation of tactile stimuli and suppression of tickliness. Blakemore et al. [21] conducted an experiment in which a device was manipulated to tickle its own hand. The results showed that the device was not ticklish under normal conditions, but when a 300 ms delay was added to the device movements, the device felt ticklish. This result can be interpreted as a delayed tactile feedback generated by the movement of the device, which is unable to attenuate the sensation by the predictions produced by the forward model, resulting in a tickling sensation.

**Figure 3:**
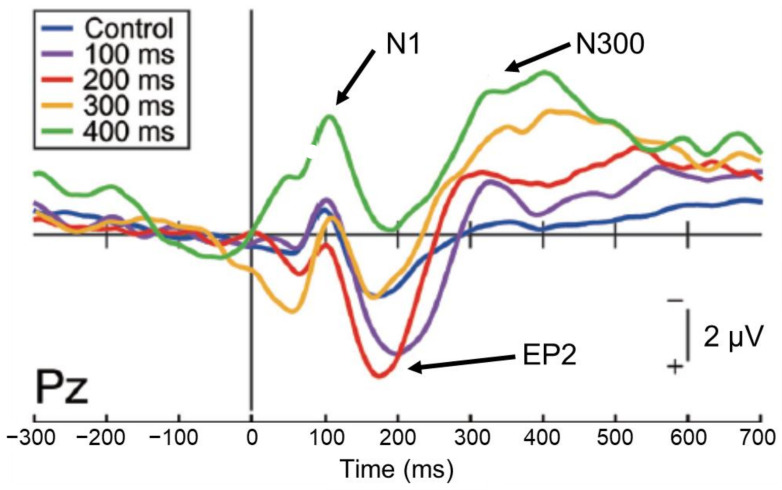
Event-related potential components for self-generated delayed sounds, related to sensory attenuation and the sense of agency [24]. The N1 component is significantly suppressed (sensory attenuation) in the control (no delay) and 100–300 ms delay conditions, but not in the 400 ms delay condition. The N300 component becomes greater as the delay increased, which reflected the attenuation of the sense of agency.

However, studies examining the direct relationship between sensory attenuation and sense of agency are still few in number and results are variable [22,23]. When brain activity was investigated by inserting a delay (100–400 ms) into the auditory feedback to self-motion (mouse clicks) to identify EEG components related to the sense of agency [24], the 50% delay discrimination threshold was approximately 160 ms. The analysis of EEG components revealed that N1 suppression occurred up to a delay width of 300 ms, but not of 400 ms (Figure 3), while the P2 component, which occurs following N1, was more active in the 100 ms and 200 ms delay conditions, close to the 50% delay discrimination threshold (Enhanced P2:EP2). This effect was attenuated at higher delays. Furthermore, the amplitude of the subsequent N300 component increased with increasing delay. The examination of the relationship between these EEG indices and the intensity of the subject’s sense of agency showed no correlation with N1 or EP2, but a significant negative correlation with N300; that is, the greater the N300 amplitude, the less the subject felt a sense of agency.

This result indicates that the sense of agency may be reflected in the subsequent late component (N300) rather than in N1 suppression; N1 suppression occurs automatically when auditory feedback of self-motion is input within 300 ms, but does not necessarily seem to directly affect the sense of agency. In addition, an enhancement of the P2 component occurs during delayed detection of auditory feedback, but this was found only in the 100 and 200 ms delay conditions, which may reflect the processing load on the stimulus. The N300 component, which arises later, finally begins to reflect conscious processes and is thought to be involved in the process that attenuates the sense of agency owing to the detection of the delay.

Intentional binding is another implicit measure of the sense of agency, and is an illusion of subjective time regarding when the intention to move is generated and when the resulting feedback occurs [25]. In the experiment, the subjects were first asked to press a button and then to listen to the sound feedback 250 ms later and then to report either the time of pressing the button or the time they heard the sound at the position of the hand on a Wundt’s clock (which makes one round in 2.56 s). Interestingly, the subjects reported that the time at which the button was pressed was later than the actual time, and that the sound feedback was heard earlier than the actual time. In trials in which the subject only presses the button or hears a sound, the subject is able to report the exact time. In addition, this effect does not occur when the subject’s finger movements are passive. In other words, the two events, button pressing and sound feedback, are felt in close temporal proximity (bound) only when button pressing is intentionally performed. This effect is called “intentional binding.” Many studies have shown that the magnitude of the intentional binding effect is related to the strength of the sense of agency: the stronger the sense of agency, the closer the perceived time of movement and sound [26].

The brain mechanism that forms the sense of agency should also be discussed. Patients with parietal lobe damage perform significantly worse on the aforementioned task of judging agency [16]. Spence et al. [27] also measured brain activity in schizophrenic patients with hallucinations and found greater activity in the right inferior parietal lobe when they reported feeling as if the movements they generated were controlled by others. Farrer et al. [28] conducted fMRI experiments with healthy subjects and found that when a virtual hand movement controlled by a joystick was spatially displaced, the activity in the inferior parietal lobe of the right hemisphere increased as the displacement increased. They further reported increased activity in the right angular gyrus when a visual feedback delay was inserted for self-generated movement [29]. Others have reported increased activity in the right inferior parietal lobe and other areas when errors in the task of moving a cursor on a screen increased [30]. A recent meta-analysis reported that bilateral temporoparietal junction (TPJ) activation is related to a decreased sense of agency [31]. Studies examining brain activity related to the sense of agency have repeatedly reported that the right inferior parietal lobe is activated when the sense of agency is disrupted. This suggests that the right inferior parietal lobe acts as a comparator in the forward model, which detects errors between the predicted and actual sensory feedback.

Balslev et al. [32] conducted an fMRI experiment in which subjects moved an on-screen cursor actively or passively and found that brain activity in the right hemisphere near the TPJ increased when hand movement was incongruent with cursor movement, regardless of whether it was active or passive. Tsakiris et al. [33] also used fMRI to measure brain activity while looking at the active or passive hand and found that the right supramarginal gyrus was activated during the incongruent condition of active movement, and the right hemisphere angular gyrus was activated during the incongruent condition of passive movement. Shimada et al. [34] also found that the inferior parietal lobe of the right hemisphere is activated during passive movements when there is a temporal delay between visual and proprioceptive feedback. These results indicate that the inferior parietal lobe of the right hemisphere is responsible for detecting the incongruence of sensorimotor and/or multisensory integration in both active and passive movements.

On the other hand, Farrer et al. [28] identified the insular cortex as a region positively associated with the sense of agency. The insular cortex is an area where proprioceptive, somatosensory, and interoceptive sensations are integrated [35]. Recent studies have reported positive activity in the insula when feeling a sense of agency [36,37,38,39]. Although further investigation is needed, these studies support the notion that the insular cortex is associated with the formation of a sense of agency.

### 2.4. Robot Hand Illusion

The rubber hand illusion can generate a sense of ownership by providing tactile stimuli to the rubber hand at the same time as one’s own hand. Similarly, the robot hand illusion (or virtual hand illusion) is the feeling of self for a robot hand that moves in accordance with the movements of the participant’s own hand. The robot hand illusion generates both a sense of agency, in which the participant feels that he or she is moving the robot hand, and a sense of ownership, in which the robot hand feels like the participant’s own hand [40].

**Figure 4:**
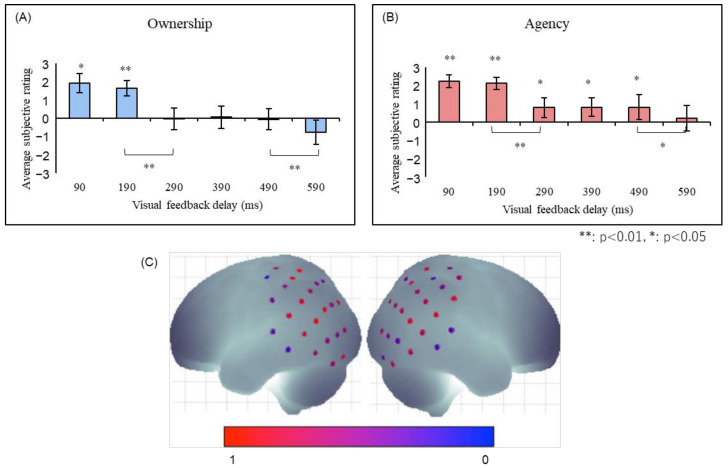
(**A**) The sense of ownership and (**B**) agency in robot hand illusion [41]. Both senses showed significant reductions in the 290-ms and 590-ms conditions, with the major difference being that the sense of agency was significantly felt in the 290–490-ms conditions, while the sense of ownership was not. (**C**) The right angular gyrus was significantly activated when the robot hand illusion was felt in the 100 ms condition compared to the 400 and 700 ms conditions [42,43].

Ismail and Shimada [41] constructed an experimental setting in which subjects wore data gloves to manipulate virtual hands using their hand movements. It was shown that both the sense of ownership and the sense of agency decayed significantly when a delay of more than 290 ms was inserted into the virtual hand movement (Figure 4). Interestingly, while the sense of ownership almost disappeared after a delay of 290 ms or longer, the sense of agency persisted, albeit attenuated. This indicates that the sense of agency is more tolerant of feedback delays and that it is possible to have a situation in which the sense of agency is felt even though the sense of ownership is disrupted. Taken together, these findings suggest that the sense of ownership requires stricter temporal consistency than the sense of agency.

The authors further measured the brain activity during the robot hand illusion using near-infrared spectroscopy (NIRS) [42,43]. In this experiment, the brain activity was assessed in the 100, 400, and 700 ms delay conditions and significantly stronger brain activity in the right angular gyrus in the 100 ms delay condition than in the 400 and 700 ms delay conditions was found. This indicates that the right inferior parietal lobe is involved in the generation of a sense of ownership and agency in the robot-hand illusion.

Currently available data showed seemingly contradictory activity in the inferior parietal lobe, being active when the sense of agency is disrupted [28,29,30,31,32] or when the sense of ownership is felt, as we have seen in the rubber hand illusion [5,9,10] and the robot hand illusion [42,43]. Further research is needed on the role of the inferior parietal lobe in the senses of ownership and agency.

### 2.5. Full-Body Illusion

The rubber hand illusion and robot hand illusion indicate that a sense of ownership or agency for the hand can also occur for objects other than self-body parts. However, it is known that such an illusion can also occur in the entire body, which is called the full-body illusion. In the full-body illusion, the back view of the subject is captured by a video camera and presented on a head-mounted display (HMD) worn by the subject. Then, tactile and visual stimuli are simultaneously applied to the subject’s own back and the back of the body seen in front of the subject (the subject’s own back). The subject eventually feels a sense of ownership of the body seen in front of them, which resembles an out-of-body experience [44,45].

There are two types of full-body illusions: the “out-of-body” type [46], in which the self-body is viewed from a slightly backward distant position, and the “body-swapping” type, in which the self and other bodies are swapped from the first-person perspective. In the body-swapping full-body illusion, a mannequin or another body is displayed on the subject’s HMD from a first-person perspective, and the subject and the mannequin’s torso are simultaneously stroked with a brush. The subject then perceives the mannequin’s body as their own body. This is realized by the same brain mechanism as in the rubber-hand illusion. It has been shown that premotor and parietal lobe networks, similar to the rubber hand illusion, are deeply involved in the body-swapping full-body illusion [47].

On the other hand, the out-of-body full-body illusion is characterized by a dissociation of the self-position from the other body in which the sense of ownership is felt, which involves a different brain mechanism than the rubber-hand illusion and the body-swapping full-body illusion [48]. To identify the brain regions that represent the change in self-location, Blanke’s group created a “mental ball drop task” and measured the extent of this divergence [46]. In this task, subjects lying down in an fMRI scanner were asked to imagine a ball falling on the ground from that position and to predict the time it would land on the ground. Prior to this task, when a full-body illusion was felt for the virtual body through the HMD in the scanner, the participants were divided into two groups: those who perceived the virtual body above them and those who perceived it below them. When the mental ball drop task was performed, the subjective drop time of the ball increased significantly in the group who reported that their body appeared to be upward, while the drop time decreased in the group who reported that their body appeared to be downward. This indicates that the time taken for the mental ball to fall depends on the body position of the subject who experienced the full-body illusion, indicating that this task is a useful measure of body position drift. We examined the brain regions that correlated with the extent of this illusion and found that activity in the left and right TPJs was significantly correlated. This suggests that the TPJ is involved in the self-position drift in external disengagement, which is consistent with Blanke et al. [49], who reported that electrical stimulation to the right TPJ in patients undergoing surgery induced an out-of-body experience. A recent study reported a correlation between the magnitude of self-location displacement and the amount of rTPJ activity in subjects who were induced to experience an out-of-body illusion through hypnosis [50].

**Figure 5:**
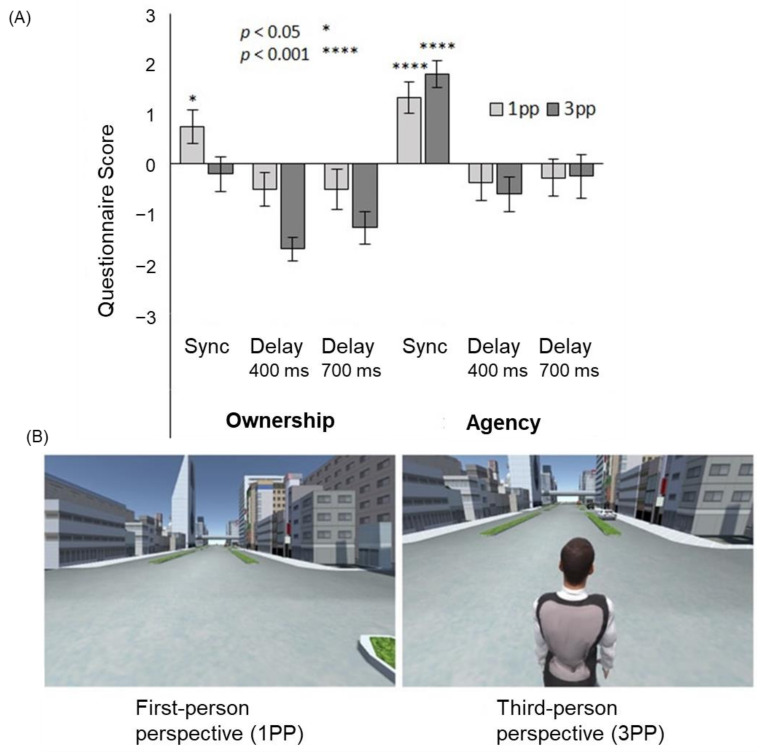
Full-body illusion from the first- and third-person perspective. (**A**) The sense of ownership was felt only in the synchronous first-person perspective (1PP) condition, while the sense of agency was felt in the synchronous 1PP and third-person perspective (3PP) conditions. (**B**) Examples of a VR scene presented to the participants [51].

In VR settings, full-body illusion can be easily accomplished by coupling the participant’s active movements with the avatar movements. Otsuka and Shimada [51] investigated the difference between the effects of first-person and third-person (body-swapping and out-of-body) viewpoints on the full-body illusion for an avatar in VR space. The subjects experienced a VR environment in which the avatar moved through the city by stepping on the spot (Figure 5). In this case, a synchronous condition, in which the avatar’s body movements matched the subject’s movement, and an asynchronous condition, in which the avatar’s movements lagged behind the subjects by 400 or 700 ms, were prepared. The results showed that the sense of agency occurred in the synchronous condition, regardless of whether it was from the first-person or third-person perspective, while the sense of ownership occurred only in the synchronous condition from the first-person perspective. These results indicate that the sense of agency is more likely to occur in the full-body illusion than in the sense of ownership, and that the full-body illusion is more likely to occur from the first-person perspective; that is, the body-swapping full-body illusion is more likely to occur than the out-of-body full-body illusion.

## 3. Back Projection and the Mirror Neuron System

### 3.1. Back Projection

Although we have described how the rubber hand illusion, the robot hand illusion, and the full-body illusion occur, several interesting phenomena occur after the illusions are generated. For example, it has been reported that when the illusion is induced for an injured rubber hand, the participant is more likely to feel pain with tactile stimulation [52]. Another study showed that when ice was placed on an illusory rubber hand, the participants felt as if their hand had also become cold, and their hand’s temperature actually decreased as well [53]. These findings indicate that the rubber hand illusion alters the subsequent sensory processing of the self-body.

Shibuya et al. [54] conducted a rubber hand illusion experiment. After the illusion occurred, the fingers of the rubber hand (actually the experimenter’s hand) were shown to open wide. It was found that when the rubber hand illusion occurred beforehand, the participant’s hand moved along with the movement of the rubber hand. Even when the participant’s hand did not move, EEG activity was observed in the motor cortex. However, no such activity was observed in the absence of the rubber hand illusion. This indicates that the participant’s hand unconsciously and spontaneously imitated the rubber hand movement after the rubber hand illusion occurred. Similar results were obtained from studies using the kinesthetic illusion, which reported that when the rubber hand in which the illusion occurred was moved, the subject’s own hand felt as if it moved in the same direction [55,56,57].

These phenomena are called “back projections” because they reflect a change in the state of the object (rubber hand), which in turn changes the state of the participant’s own hand. By projecting one’s own body image onto the rubber hand, an inverse (from the rubber hand to the participant’s body) influence has taken place. Interestingly, while the rubber hand illusion is caused by the integration of visual and tactile information, back projection is extended not only to tactile sensation but also to pain, cold sensation, and body movement. This implies that the rubber hand illusion is not merely a mapping between vision and touch, but rather a transformation of the self-body image and schema in such a way that the rubber hand becomes a part of the self-body.

Some medical applications of back projection have been proposed, including a rehabilitation method that applies the virtual hand illusion to patients with movement and pain disorders, which has been reported to be effective [58,59,60,61]. Rehabilitation methods that use back projection to correct negative body images that cause pain are well worth further testing.

### 3.2. Mirror Neuron System and Body Image

Now, I would like to step back from self-recognition for a while and look at how self-body image is involved in the recognition of others.

**Figure 6:**
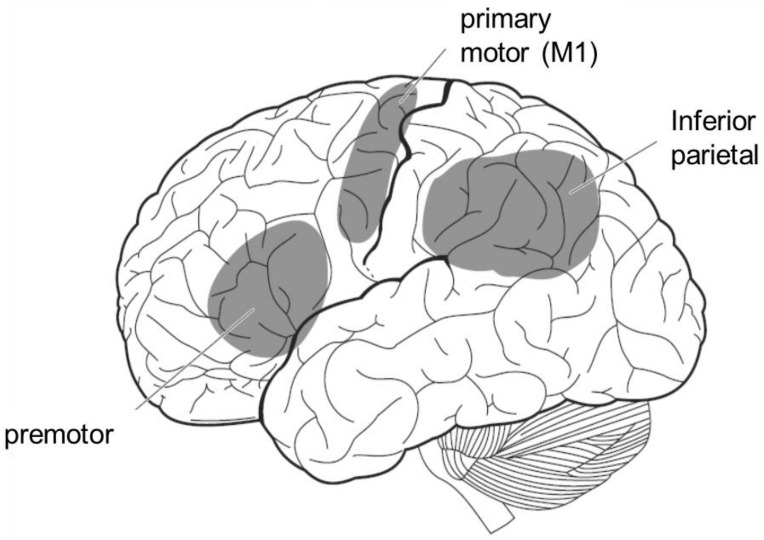
The mirror neuron system. It is activated not only when the individual performs a certain action, but also when he or she observes others performing the same action.

The mirror neuron system is a term for brain regions that are active not only when the individual performs an action, but also when the individual observes that others perform the same action. In humans, it includes the premotor area, the inferior parietal lobule, and the primary motor cortex (Figure 6). Since these brain areas are involved in motor control, it is reasonable to assume that they are activated when an individual performs an action. However, it is interesting to note that motor-related brain activity is triggered by mere observation of another person’s action, even though the individual is not performing the action. This implies that the visual input of another person’s action is converted into a motor representation of the self.

One of the major hypotheses for the cognitive functions that the mirror neuron system is responsible for is the “simulation hypothesis”, in which the mirror neuron system is used to understand the intentions and feelings of others at a deeper level [62,63]. The fact that one’s own motor cortex is activated by watching another’s movement suggests that one simulates the other’s movement using one’s own motor representations in the brain. This allows the observer to understand the intentions of why the other makes the movement and how they are feeling at the time, and to “experience” it at a deeper level than mere visual analysis. The simulation hypothesis suggests that one’s own body image is deeply involved in the recognition of others.

A vast amount of previous research has shown that the mirror neuron system is not equally active whenever we observe others’ movements. The intensity of its activity varies depending on various factors (for a review, see Ref. [64]).

For example, previous studies have shown that the mirror neuron system selectively modifies its activity, depending on the extent to which the movement is familiar to the observer. Calvo-Merino et al. [65] measured the brain activity of dancers specializing in relatively similar but different types of body movement (ballet and capoeira). They found that the mirror neuron system was significantly more activated when dancers specializing in ballet watched ballet and when dancers specializing in capoeira watched capoeira than when they watched another movement. These results indicate that the mirror neuron system is more strongly activated when observing movements that are part of one’s own motor repertoire than when observing movements that are not.

On the other hand, many studies have shown that the mirror neuron system is activated selectively on the motor intentions of others rather than on the kinematic details of the movement [66,67,68]. For example, Umilta et al. [66] demonstrated to monkeys a grasping movement in which the experimenter reached out with their right hand to grasp an object on a table. As expected, the monkeys’ mirror neurons were activated by observing the actions of the experimenter. Next, they showed the monkey the object on the table and then placed a screen in front of it so that the monkey could not directly see the object. Under these conditions, when the grasping movement was performed, the mirror neurons were activated at the exact moment when grasping took place, even though the monkeys could not see the scene of grasping directly. Finally, when the monkeys were shown a similar movement after being shown nothing behind the screen, the mirror neurons were not activated. These results suggest that the monkeys understood the experimenter’s movements while “imagining” that the experimenter was actually grasping an object.

Another possible function of the mirror neuron system is the imitation of the motion of others. To imitate, one must generate one’s own motor output from the visual information of the other’s action. Iacoboni et al. [69] reported that the mirror neuron system is more active when imitating an action of someone else than when performing the same action using other visual stimuli as cues. Vogt et al. [70] also reported that the mirror neuron system was more active when the participants were asked to imitate the action of pressing a guitar chord and observed others’ hands to imitate the chord pressing they had not practiced before than when they were asked to press a chord they had practiced. These results indicate that the mirror neuron system activity is greater when observing others’ movements with the intention to imitate them, and thus indicate a link between the mirror neuron system and imitation.

Finally, recent studies have investigated the involvement of the mirror neuron system in cheering. In a study conducted by the authors, subjects viewed videos of two players playing rock-paper-scissors and were asked to watch one of the players while cheering. The activity of the mirror neuron system was measured using NIRS. When the cheered-for player won, the mirror neuron system was more active than when the player lost or when the two players drew [71]. Furthermore, this activity was stronger when the cheered-for player was presented in the same orientation as oneself (first-person perspective) than in the opposite orientation (third-person perspective) [72].

Shimada et al. [73] conducted an fMRI experiment to investigate the association between the mirror neuron system and the reward system during cheering. The results showed that the mirror neuron system (premotor cortex) was activated while observing the player’s actions, which was significantly correlated with the strength of the sense of unity with the player. In addition, the ventromedial prefrontal cortex (vmPFC), which is the main component of the reward system, was more strongly active, and the functional connectivity between the mirror neuron system and the vmPFC was significantly greater in the condition in which the cheered-for player succeeded than when they failed. These differences were not observed in players who were not cheered.

**Figure 7:**
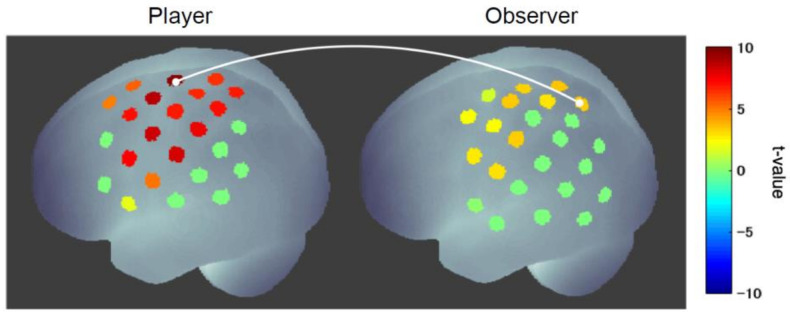
Functional connectivity between player and observer in cheering [74]. The connectivity between the player’s motor cortex and the observer’s mirror neuron system was significantly enhanced when the player won compared to when the player lost.

Next, a hyperscanning (simultaneous measurement of two participants) experiment was conducted to examine the functional connectivity between the player’s and observer’s brains and found strong connectivity between the player’s motor cortex and the observer’s mirror neuron system (superior parietal lobule) (Figure 7) [74]. This coupling was stronger when the player won than when he lost and was significantly stronger in the cheering group than in the control group (judging whether the player cheated or not). Furthermore, the strength of this connectivity was significantly correlated with the scores for the sense of unity that observers in the cheering group felt toward the player. These results indicate that a sense of unity between player and observer is formed by cheering and that the degree of unity can be inferred from the strength of the functional connectivity between the player’s and observer’s internal motor activities.

### 3.3. Mirror Neuron System and Back-Projection

As seen in the previous section, the mirror neuron system activity varies depending on various factors. The fact that the mirror neuron system is active when we are watching a movement that is part of our own movement repertoire, a movement for which we understand the intention of the other, a movement that we are trying to imitate, or a movement that we cheer for means that the intensity of the mirror neuron system activity reflects how we actively simulate the movement of others using our own body image, which is considered to be a state of incorporation of the other into self. This may be very close to the state of identification of a rubber hand with one’s own body in the rubber hand illusion.

Previous studies on back projection showed that when a movement of the rubber hand occurs, a similar self-body movement is induced automatically (see Section 3.1). Similarly, in the case of the mirror neuron system, the observation of the other’s body movements elicits motor activation of the self. Thus, the activity of the mirror neuron system can be considered a special case of back-projection. That is, when the self-body image is projected onto the “other’s body”, rather than the “rubber hand,” the other’s body movement is back-projected onto the self-body. Thus, the mirror neuron system activity is likely to be a back-projection resulting from the extension of the projection to the other’s body. This is also confirmed by the fact that the brain areas involved in the rubber hand illusion and the mirror neuron system are the frontoparietal sensorimotor network, which are nearly identical, suggesting that the brain mechanisms for both are the same (Table 1).

In this sense, it can be said that our self-body image is not limited to our own body but is also open to others. Self-body illusions, such as the rubber hand illusion, are projections of the self-body image onto other body-like objects that match the multisensory or sensorimotor information of the self-body. In the case of the mirror neuron system, one’s own body image is projected onto the other’s body. In any case, the self-body image has a mechanism to actively incorporate others’ bodies when possible and appropriate, and in this sense, the mechanisms of the self-body illusion and the mirror neuron system can be considered to be fundamentally the same.

## 4. Multisensory and Sensorimotor Integration in the Embodied Self

To conclude this paper, I will discuss the relationship between self-body recognition and the mirror neuron system. In other words, there is a relationship between the neural mechanism that discriminates between the self and others and the neural mechanism that fuses the self and others. As we have seen, these brain areas actually overlap to a considerable extent, and in both cases the key areas are the sensorimotor-related areas in the frontal and parietal lobes.

**Figure 8:**
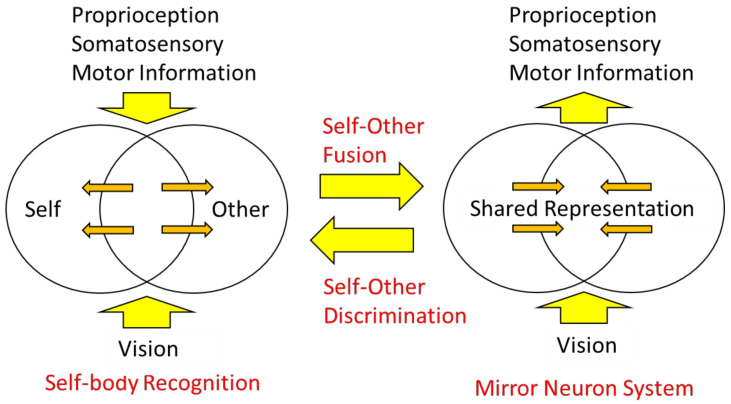
A model for self-body recognition and mirror neuron system. In self-body recognition (**left**), the vision (extrinsic body information) is compared with the intrinsic (proprioceptive, somatosensory, or motor) body information. In the mirror neuron system (**right**), extrinsic body information drives intrinsic, especially motor, body representation.

Let us now consider two types of body information: extrinsic body information derived primarily from visual and auditory inputs and intrinsic body information derived from sensory inputs and motor commands. The mirror neuron system is considered a brain mechanism in which the intrinsic body responds to the extrinsic body information (Figure 8, right), that is, sensory-to-motor transformation. On the other hand, in self-other discrimination, extrinsic body information is compared with intrinsic body information, and when the two are consistent, the self-body image becomes active, and when they are not, processing of the visual body as the other’s body is initiated (Figure 8, left), which is underpinned by multisensory integration.

**Figure 9:**
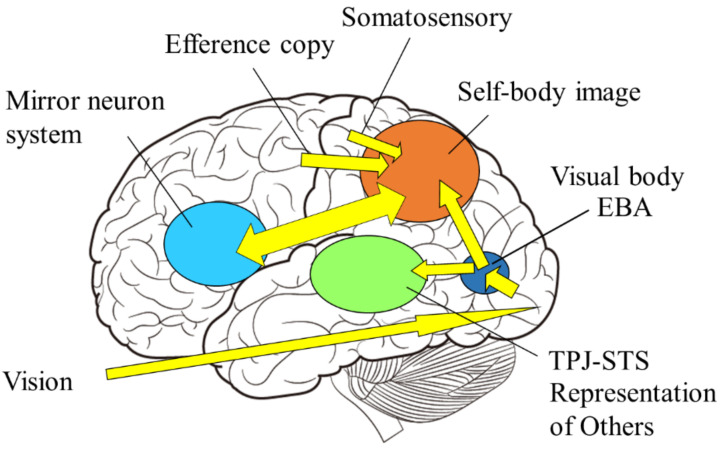
Self-body recognition and the mirror neuron system. Extrinsic (vision, sound) and intrinsic (proprioceptive, somatosensory, and motor) body information are integrated in the parietal lobe. If those are consistent, the self-body image emerges in the superior parietal and intraparietal regions, and possibly extends to the premotor cortex, consisting of mirror neuron system, otherwise representations of others emerge in the TPJ and/or STS regions.

In other words, the mirror neuron system issues the motor command to minimize the difference between the intrinsic and extrinsic bodily information, while the function of self-other discrimination is to check the consistency among the intrinsic and extrinsic multisensory bodily information. The author proposes the following model as a neural mechanism to realize such dual functions (Figure 9): first, visual stimuli related to the body are input into the visual cortex in the occipital lobe and identified as body parts (external body representations). The brain area in the visual cortex, called the extrastriate body area (EBA), selectively responds to visual body input [75]. On the other hand, sensory information from somatosensory areas and motor information derived from motor areas (intrinsic body representations) are projected to the parietal lobe. In the parietal lobe, the integration and matching of extrinsic and intrinsic body information is performed based on spatiotemporal consistency. As a result of the matching, the extrinsic body that is consistent with the intrinsic body is recognized as the self-body, and the senses of ownership and agency are generated. In contrast, the extrinsic body, for which spatiotemporal inconsistency is detected, is processed in the inferior parietal lobe or TPJ and perceived as the other’s body.

On the other hand, the mirror neuron system is considered as follows: first, as before, the extrinsic body is identified in the visual cortex and its consistency with the intrinsic body is examined. At this point, the difference does not rise to consciousness as it does when we distinguish between the self and others, but rather, the intrinsic body is automatically adjusted (i.e., motor commands are automatically generated) to eliminate the difference. This is plausible when considering that movement is almost unconsciously controlled based on visual feedback (e.g., online motor control using visual feedback). This mechanism explains how mirror-like self-movements can be triggered by watching another person’s movement.

It is important to note that the mirror neuron system does not transform the other’s actions into self-actions. The mirror neuron system is defined as (1) active when the individual performs a movement and also (2) active when the individual sees another person performing the same movement. If its functional role is “transformation”, then it does not need to fulfill point (1) of the above definition in the first place. The mirror neuron system is only “evidence” that others and the self are mapped, not the mechanism. The existence of a mirror neuron system indicates that the visual input of another person’s body can drive or reference the motor representation of the self. What this fact shows is that the representations of the body in the brain are not limited to “my” own body, but that the visual input of other bodies can also evoke the intrinsic body of the self, and that our body is open to others through the medium of vision.

The key to understanding how we can understand others is not in the superficial behavior of the mirror neuron system, but rather in how the extrinsic and intrinsic bodies interact and are integrated into the body image or body schema. By considering the mechanism of multisensory and sensorimotor integration regarding the self and others’ bodies, in other words, the dynamics of body representation in the brain, we can comprehensively understand the two seemingly contradictory brain functions of self-body recognition and the mirror neuron system, and hence the neural mechanism of the embodied self.

## Figures and Tables

**Table 1:** Brain regions involved in self-body recognition and MNS.

Experiment	Function	Brain Region
RHI	SoO	premotor [5,9,10,11]
	Proprioceptive drift	IPL [11]
SoA	Sensory attenuation	sensory (auditory) cortex [19,20,24]
	Comparator	IPL, TPJ [28,29,30,31,32,33]
RoHI	SoO & SoA	IPL [42,43]
FBI	Out-of-body experience	TPJ [48,49,50]
MNS	Action observation	IPL, SPL, premotor, M1 [62,63,64,65,66,67,68,69,70,71,72,73]

RHI: rubber hand illusion, SoO: sense of ownership, SoA: sense of agency, RoHI: robot hand illusion, FBI: full body illusion, MNS: mirror neuron system, IPL: inferior parietal lobule, TPJ: temporo-parietal junction, SPL: superior parietal lobule, M1: primary motor cortex.
